# Prospective comparison of a PCR assay and a microbiological culture technique for identification of pathogens from blood and non-blood samples in septic patients

**DOI:** 10.1186/s40560-015-0116-1

**Published:** 2015-11-21

**Authors:** Runa Plettig, Andreas Nowak, Veronika Balau, Klaus Hahnenkamp, Taras Usichenko

**Affiliations:** Department of Anesthesiology, Intensive Care, Emergency and Pain Medicine, Hospital Dresden-Friedrichstadt, Dresden, Germany; Friedrich Loeffler Institute of Medical Microbiology, University Medicine of Greifswald, Greifswald, Germany; Department of Anesthesiology, Intensive Care Medicine, Emergency Medicine and Pain Medicine, University Medicine of Greifswald, Greifswald, Germany

**Keywords:** Sepsis, Molecular-based diagnostics, Microbiological culture

## Abstract

**Background:**

Molecular amplification techniques are suggested to be a useful adjunct in early detection of pathogens in septic patients. The aim was to study the feasibility of a polymerase chain reaction (PCR) assay compared to the standard microbiological culture (MC) technique in identification of pathogenic microorganisms from blood and non-blood samples in septic patients.

**Methods:**

Samples for pathogen identification were taken during febrile septic episodes (SE) in 54 patients with sepsis and analyzed using both MC and PCR. Semi-automated multiplex PCR, provided by Philips Medical Systems, was able to detect nine different pathogens. The accuracy of pathogen identification using PCR vs. MC as well as the time-saving effect of PCR on the potential decision-making process for antimicrobial therapy was evaluated.

**Results:**

In a total of 258 samples taken during 87 SE, both methods yielded more pathogens from the non-blood than blood samples (87 % vs. 45 %; *p* = 0.002). PCR identified more pathogens than MC in the blood samples (98 vs. 21; *p* < 0.0001), but not in other body fluids. In 35 SE, the potential decision on appropriate antimicrobial therapy based on PCR results could have been made 50 (median; interquartile range 35–87) hours earlier than decisions based on standard MC.

**Conclusions:**

In septic patients, multiplex PCR identified more pathogenic microorganisms isolated from the blood samples than the standard MC technique. In the non-blood samples, PCR was comparable to that of MC.

**Electronic supplementary material:**

The online version of this article (doi:10.1186/s40560-015-0116-1) contains supplementary material, which is available to authorized users.

## Background

Sepsis is a common infectious cause of morbidity, requiring intensive care measures and immediate effective antimicrobial therapy. Despite extensive therapeutic options, mortality rates range from 10 to 20 % in patients with uncomplicated sepsis and up to 80 % in patients with septic shock [1], ranking sepsis as the most common cause of death in non-cardiac intensive care units [2].

The surgical removal of septic foci and an early adequate administration of antimicrobial treatment dramatically improve the clinical outcome of septic patients [3]. Inadequate initial antibiotic treatment significantly increases the mortality rate [4]. Furthermore, delay in administration of effective antimicrobial treatment increases mortality by the hour [5, 6]. Prompt identification of the causative pathogen and of its antimicrobial resistance pattern is of crucial importance for effective treatment of sepsis [5].

The microbiological culture (MC) technique is the conventional “gold standard” method for the identification of bacterial and fungal infections in patients with sepsis. However, sepsis diagnostics using microbiological culture is possible only with viable pathogens. Their growth time requires up to 48 h to yield the final result, which may be negative in up to 30 % of cases [7, 8].

Pre-treatment with antibiotic or antimycotic agents has a negative impact on the growth of the causative pathogen [9]. Nonetheless, despite a low sensitivity [10], the positive results in blood culture guarantee the identification of the causative pathogen and its phenotype of antimicrobial resistance, which is required for successful treatment.

In recent decades, polymerase chain reaction (PCR)-based molecular amplification techniques have been suggested as a promising diagnostic tool for a faster identification of sepsis-causing pathogens [11–13], whereby only blood samples are taken for sepsis diagnostics.

The aim of our investigation was to study the feasibility and accuracy of a PCR assay compared to the standard microbiological culture technique for detection of pathogens in blood samples and samples of other body fluids (bronchial secretions, wound fluid, abscess fluid, smears, etc.) in patients with sepsis. In addition, we wanted to evaluate the potential time-saving effect of PCR on the decision-making process for the initiation of antimicrobial treatment.

## Methods

### Patients and study design

This single-center investigation was performed at the surgical intensive care unit of the tertiary hospital with a capacity of 900 beds. The local ethics commission approved the investigation; the consent of the patients for this observational study was not needed. All patients older than 18 years of age with a known or suspected focus of infection, and at least two clinical signs of systemic inflammatory response syndrome (SIRS), were included in the study. Diagnostic criteria for SIRS, sepsis, severe sepsis, and septic shock were defined as proposed by the expert committee of the American College of Chest Physicians and the Society of Critical Care Medicine (ACCP/SCCM 1992) [14]. Patients with SIRS without a septic focus or with a confirmed acute viral infection were not included in the study.

This was a prospective observational laboratory and clinical investigation. Blood samples and specimens of other body fluids were taken from the suspected septic foci in patients enrolled in the investigation according to the above-described criteria. All samples were collected using sterile technique for analysis of potential pathogens. The analysis was performed simultaneously using standard MC techniques and a multiplex PCR procedure. The anti-infective therapy of septic patients, which was initially started as empiric treatment according to current guidelines [15], was changed further only on the basis of the MC diagnostics; the results of the PCR analysis were not disclosed to the attending physicians, so the results of PCR diagnostics did not influence the therapy of patients with sepsis, included in this investigation.

### Sample collection

Blood sample collection was performed according to microbiology procedure quality standards (MIQ) [16]. The blood samples were taken at septic episodes (SE), when pyrexia, hypothermia, or chills were recorded. Septic episodes were defined according to ACCP/SCCM sepsis definition [14]. If, despite anti-infective therapy, the fever persisted, or if an increase of body temperature or an increase of infectious parameters (procalcitonin, C-reactive protein) occurred, the blood samples were collected anew. At least 20 ml of blood was collected per septic episode. Aerobic and anaerobic blood culture bottles (BACTEC® PLUS™ Aerobic/F and Anaerobic/F, Becton Dickinson Diagnostic Instrument Systems) were inoculated with 9 ml of blood per bottle. The blood (2 ml) was inoculated in an EDTA-Monovette® (Sarstedt) for PCR analysis. Blood culture bottles were incubated for a maximum of 9 days in a continuously monitored incubator (BACTEC 9240; Becton Dickinson) at 37 °C.

Sample collection from other body fluids occurred as ordered by the attending physician and as clinically indicated according to the suspected septic focus. Samples included tracheal and bronchial fluid, abscess and drainage fluid following surgical debridement, peritoneal fluid, cerebral fluid, urine samples, and smears of wounds. To allow simultaneous analysis with the two different techniques, the samples were split in two under sterile conditions. Samples for PCR analysis were stored at 7 °C before processing. Samples for analysis using MC were transported to the in-house microbiological laboratory and retained until processing (depending on laboratory working hours) according to in-house standards for microbiology procedures (e.g., wound smears). Smears were collected in cases where the body fluid collection was not possible: two swab samples per infected area were taken in order to allow analysis with both MC and PCR techniques.

### Multiplex PCR

To conduct the multiplex PCR, an experimental arrangement of the commercial lab apparatus, designed and provided by Philips Medical Systems (PMS) Böblingen, Germany, was used. Sample processing and analysis occurred in five steps. Step 1: cell lysis and DNA extraction were achieved using EZ1® cartridges (Qiagen, Venlo, Holland); cells in the samples were destroyed by mechanical and chemical processes, and the released DNA was bound to magnetic particles, washed several times, and then eluted in water. Step 2: the extracted DNA was incubated with specific primers in QIAGEN Multiplex PCR Mastermix. Multiplex PCR Mastermix consisted of HotStarTaq DNA Polymerase, QIAGEN Multiplex PCR Puffer, 5× concentrated Q-solution, the dNTP Mix, and RNase-free water. Both of these steps were performed on the fully automated EZ1 BioRobot® platform (QIAGEN). Step 3: a thermocycler (PCR System 9700, ABI GeneAmp, LifeTechnologies GmbH, Darmstadt, Germany) was used for DNA amplification. Step 4: the pathogen’s (bacterial and fungal) DNA was detected by means of electrophoresis with “lab on a chip” technology (DNA 1000 LapChip® Kit, Agilent Technologies, Frankfurt, Germany). Step 5: the isolated pathogen DNA was identified by comparison with a DNA ladder (BioAnalyzer 2100, Agilent Technologies). Dilution of samples other than the blood occurred on a 1:5 or 1:10 basis using isotonic saline solution depending on the viscosity of the sample fluid. If the PCR analysis recorded high amounts of noise, analysis was repeated in further dilution steps (1:50, 1:100). The samples for PCR diagnostics arrived to the lab at latest in 60 min following the samples collection and were immediately processed.

Specific primers were developed by PMS for identification of the causal microorganisms. The collected patient samples were analyzed for the presence of nine frequent sepsis pathogens: *Staphylococcus aureus*, *Staphylococcus epidermidis*, *Enterococcus faecium*, *Enterococcus faecalis*, *Escherichia coli*, *Klebsiella pneumonie*, *Enterobacter cloacae*, *Pseudomonas aeruginosa*, and *Candida albicans*.

### Microbiological culture technique

The MC techniques were carried out at the microbiological laboratory of the Dresden-Friedrichstadt hospital according to the in-house standards. Positive blood culture samples and all non-blood samples were subject to microscopic analysis using Gram staining. The attending physicians were informed by a telephone call when microscopic analyses revealed the presence of microorganisms. All samples were cultivated under aerobic and anaerobic conditions and standard incubation temperatures depending on sample origin and suspected pathogen using blood culture machines BACTEC 9240 (Becton Dickinson, USA). The blood samples were incubated at 37 °C for max. 9 days. In case if the living microorganisms were present in the blood, their growth caused the increase of CO2 in the blood culture bottles. This increase was measured using the increase of fluorescence by chemical sensor, which delivered both optical and acoustical signal. The precise identification of the bacterial pathogens was performed using the BD Phoenix™ Automated Microbiology System (Becton Dickinson, USA). Analysis for growth of microorganisms was performed after 24 h of incubation and further analysis after 48 and 72 h. The first microbiological laboratory findings were reported within 24 h. Standardized identification and susceptibility tests were usually available after 48 h. Within working hours, the samples reached the microbiological laboratory in 30–60 min; if the samples were taken in the night or over the weekend, the delay to arrival to the lab could be maximally 16 h.

### Samples evaluation and data analysis

The pathogen was considered as true positive (causal for infection) if (i) this pathogen was found simultaneously in two separate samples obtained from the same patient using either of the detection techniques (MC or PCR), (ii) the pathogen count, which was estimated semi-quantitatively using the MC technique, was higher than “moderate quantity” (Additional file 1), (iii) concentration of DNA of potential pathogen, which was amplified in PCR, was higher than 0.2 ng/μl (in cases where the identified microorganism in two separate samples could present as a part of the physiological bacterial flora (in case of non-blood samples), DNA concentrations greater than 10 ng/μl were considered as a relevant pathogen), and (iv) the clinical picture of infection corresponded to the results of laboratory diagnostics. Conversion charts are proposed in order to compare the results of the diagnostic techniques (Additional file 1).

The test results of both techniques were compared and evaluated with reference to positive rates of samples and the concordance of type and quantity of the detected pathogens. To study the accuracy of the methods, the test results were compared to the results of other accompanying investigation tests (blood or non-blood material). The chi-square test was applied for dichotomous data where appropriate. Furthermore, the time from the sample collection to disclosure of test results was determined for both methods. The potential time-saving effect was calculated as the difference between the time required for pathogen identification using MC technique and the time required for pathogen identification using PCR.

## Results

### Patients

**Fig. 1:**
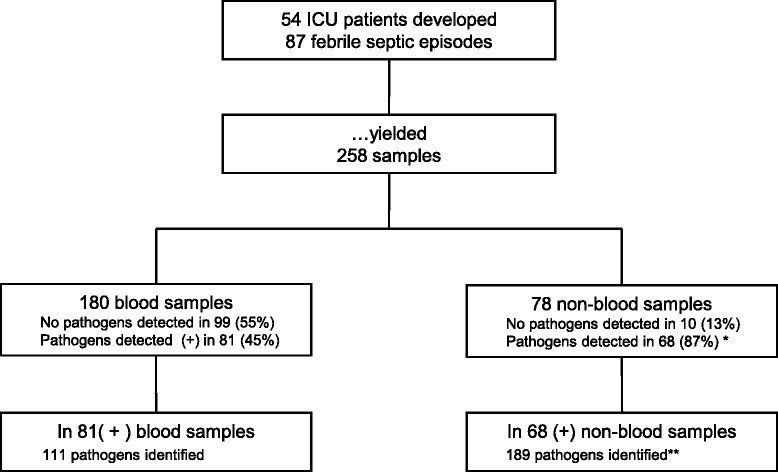
Flow chart of the study with the number of pathogens, detected by both microbiological culture technique and multiplex PCR in patients with sepsis at the intensive care unit (ICU). Both diagnostic techniques yielded more positive results and detected more septic pathogens in non-blood samples as compared to blood samples. *p = 0.002; **p = 0.001; chi-square test

A total of 54 patients with clinical diagnosis of sepsis were included in this study. The median age was 67 years (range, 21–91 years). These patients developed 87 febrile septic episodes, of which 4 were categorized as sepsis, 42 as severe sepsis, and 41 as septic shock (Table 1). In total, 258 samples (180 blood samples and 78 samples of other body fluids) were collected for analysis (Table 2, Fig. 1), whereas 3 samples per patient per SE were collected on average.

**Table 1:** Septic episodes differentiated according to the site of suspected focus and sepsis severity

Septic focus	Severity of sepsis			Total
	Sepsis	Severe sepsis	Septic shock	
Abdomen	1	8	11	20
Abscess	0	5	4	9
Central venous catheter	1	10	12	23
Lung	0	11	13	24
Genitourinary tract	1	2	0	3
Infected wound	1	4	0	5
Other	0	2	1	3
Total	4	42	41	87

Data are given as a number of septic episodes

**Table 2:** Number of the samples for pathogen identification according to their origin

Material	Number
Blood	180
Bronchial secret	34
Wound fluid	16
Abdominal lavage fluid	8
Smears	6
Urine	5
Abscess fluid	3
Puncture fluid	3
Cerebrospinal fluid	3
Total	258

### Blood vs. non-blood samples

Both MC and PCR techniques detected pathogens, which caused sepsis, in 45 % of the blood samples vs. 87 % of the non-blood samples (*p =* 0.002; Fig. 1). One hundred and eleven pathogens were identified by both diagnostic techniques in 81 blood samples vs. 189 pathogens from 68 non-blood samples (*p* = 0.001; Fig. 1).

**Fig. 2:**
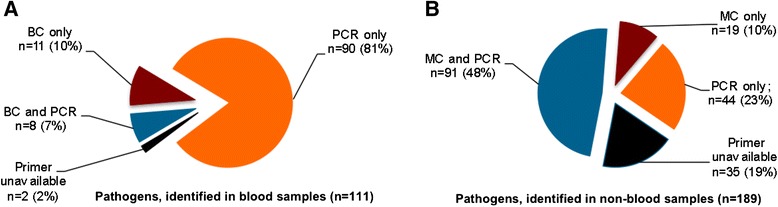
Identification of septic pathogens from blood (**a**) and non-blood samples (**b**) using a microbiological culture (*MC*) technique and multiplex PCR (*PCR*). Values are absolute numbers (percentage) of identified pathogens. *p = 0.0002; chi-square test

**Fig. 3:**
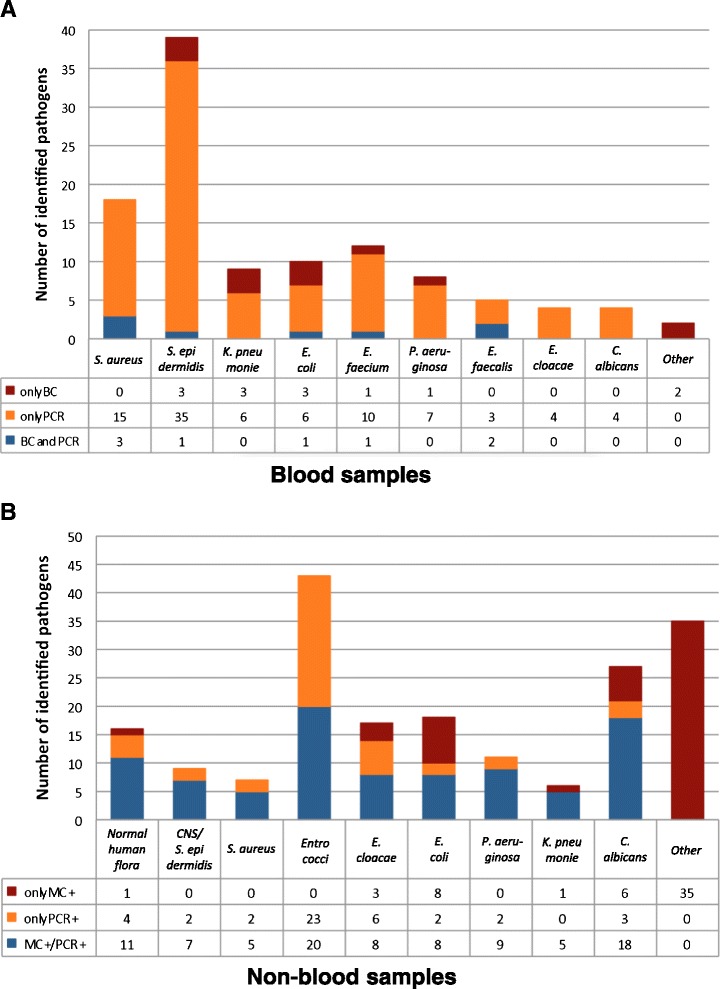
Number of various pathogens identified in **a** blood samples and **b** non-blood samples using microbiological blood culture technique and PCR

In the blood samples, PCR identified more pathogens than MC (98 vs. 21; *p* < 0.0001), whereas the yield of both techniques from the non-blood samples was comparable (PCR 135 vs. MC 145, Fig. 2). In 2 (2 %) cases, the pathogens could not be identified by PCR in the blood samples due to the lack of primers vs. 35 (19 %) cases in the non-blood samples (*p* = 0.0002; Fig. 2a). Detailed description of pathogens identified by both methods is given in Fig. 3. Forty-six of the 90 pathogens identified by PCR were confirmed by detection in several blood samples taken at the same time. Additional 24 pathogens were confirmed by means of PCR or MC in other samples such as bronchial secrets, urine, abscess, tip of central vein catheter, etc. In 20 pathogens, the DNA concentration to be identified by PCR was higher than 0.2 ng/μl. *S. epidermidis*, detected in 35 cases only by PCR from the blood samples, was responsible for 14 septic episodes clinically associated with central venous catheter infection.

### Septic episodes

Both PCR and MC technique yielded negative results in 20 % of septic episodes and could detect the pathogens in 46 % of 87 SE (Table 3). MC yielded positive results (and PCR failed) in 3 % of SE, whereas PCR identified organisms that were not detected by MC in 31 % of SE (*p* = 0.001). Clinical details of SE where MC failed and PCR could identify the septic pathogens are presented in Additional file 2.

**Table 3:** Accuracy of pathogen detection during septic episodes using PCR and microbiological blood culture (MC)

Septic episodes (*n* = 87)	MC (−)	MC (+)	Total
PCR (−)	17 (20)	3 (3)	20 (23)
PCR (+)	27 (31)	40 (46)	67 (77)
Total	44 (51)	43 (49)	87

Data given as number (percent) of cases

In four cases, the relevant pathogens, identified by MC, were not recognized in the study samples by PCR because the primers were not available. In another six cases, relevant pathogens were not recognized by means of PCR even though specific primers were used.

MC and PCR techniques identified the same pathogens in 35 SE. In 12 of them, the initial empiric anti-infective therapy was confirmed by both methods and continued. In 23 SE, the initial anti-infective therapy was changed according to the results, which were confirmed by both methods. During the diagnostics within these 35 SE, PCR was faster than MC in identification of pathogenic microorganisms: the potential time-saving effect of PCR was 50 (median; interquartile range 35–87) hours (Additional file 3).

## Discussion

In this prospective investigation, a multiplex PCR assay was feasible for the identification of pathogenic microorganisms along with the standard MC technique in patients with sepsis at the surgical intensive care unit. In almost all septic episodes, both techniques confirmed the clinical diagnosis of sepsis; none of clinical diagnoses of sepsis was disapproved by these laboratory techniques. Both PCR and MC techniques identified more causative pathogens from the non-blood than from the blood samples in septic patients, whereas PCR identified more pathogens from the blood samples compared to standard MC. This fact was restricted to the diagnostics from the blood, but not from the non-blood samples; in latter comparison, both diagnostic methods were equally effective. Under clinical conditions, PCR required less time than MC for identification of causative pathogens from both the blood and non-blood samples taken during SE in patients with sepsis. Regarding the reports from other research groups comparing PCR and MC techniques, this finding was well expected [11–13, 17, 18]. In 35 septic episodes, the PCR identified the causal pathogens 50 h earlier than the standard MC. In 27 SE, the microbiology failed, and relevant pathogens were solely detected by the use of PCR. These findings suggest the clinical improvement in the identification of causal septic pathogens and thus earlier initiation of causal anti-infective therapy in the near future.

The higher diagnostic yield by both MC and PCR techniques in the non-blood samples vs. the blood samples can be explained by the higher concentration of intact viable pathogens in the non-blood samples, which were not exposed to antibiotic agents [19].

The low sensitivity of MC of the blood samples in our study confirmed the results of previous investigations, where the causative pathogens of bacteremia in septic patients could be identified in 6–23 % (increased to 69 % in septic shock) of cases using the standard MC procedure [20, 21].

However, the positive rate of PCR technique with 98/180 vs. MC with 21/180 was relatively higher (almost 5:1) compared with those reported in previous studies using multiplex PCR system. So Yanagihara et al. demonstrated that SeptiFast PCR kit identified 24 pathogens vs. 11 by MC technique out of 400 blood samples of septic patients [11]. This discrepancy in the rate of pathogen detection, which was even lower (2:1) in other investigations [12, 19], can be explained by selection of patients: in contrast to previous studies from internal medicine departments, where approximately 70 % of patients revealed SIRS and uncomplicated sepsis [11, 12, 19], almost 90 % of patients from our investigation were in severe sepsis and septic shock. Thus, it is possible that the blood samples of our patients from surgical intensive care unit, who were already pre-treated with antieffective drugs, contained high concentration of non-viable pathogens, which was detected by PCR but not by MC technique.

The detection of mainly staphylococci in 27 SE only by PCR, where MC failed, might represent a false positive result. However, in our investigation, the detection of *S. aureus* and *S. epidermidis* in 20 SE was correlated with the clinical presentation of septic patients, where 19 out of 27 SE were diagnosed FUO and 1 with catheter-associated infections. This finding is in agreement with previous reports, where staphylococci were found to be the cause of FUO in more than 50 % of cases [22, 23].

Our finding that the results of PCR and MC diagnostics of septic pathogens from the non-blood samples are comparable is in contrast with the results of Mencacci et al., who demonstrated that the commercial PCR-based system SeptiFast yielded more positive results than MC (49 % vs. 19 %; *p* = 0.001) in the detection of microbial pathogens from cardiac valve tissues and synovial and other purulent body fluids [24]. This divergence in results can be explained by (i) different sample sources—in our study, the non-blood samples were taken mainly from the bronchial secretions and wound and abdominal lavage fluids—and (ii) the broader spectrum of SeptiFast PCR, which is able to identify 25 microbial agents [13, 22].

The known limitations of the MC technique, such as poor sensitivity, time dependence, and false-negative results under antibiotic therapy [7–10, 17–21] require the development of rapid reliable methods of pathogen identification such as PCR-based diagnostics. However, PCR also reveals certain limitations. The detection of pathogen DNA is dependent on the availability of specific primers [25]; in this study, we analyzed the samples for the presence of only nine common sepsis pathogens. Thus, two pathogens were not recognized in the blood samples and 35 pathogens in the non-blood samples by PCR due to lack of primers. Moreover, contamination is a further problem of PCR-based diagnostic methods, since they detect the pathogens only if specific DNA segments are present, even from non-viable or already phagocytized pathogens [26, 27]. On the other hand, the PCR technique may yield false-negative results as well. In our study, 11 pathogens were not identified in the blood samples and 19 pathogens in the non-blood samples by means of PCR even though suitable primers were used. Deficient primers, genetic mutations of the pathogens, cross-reactions with other DNA segments, or unstable connections of the primer to the DNA might be the cause [13, 17].

The limitations of the present feasibility investigation, performed in single institution, include the small number of the microorganisms, which could be identified by PCR technique, as well as the small number of patients with sepsis, included in this study. Moreover, due to observational design of the study, we can only postulate the clinical significance of the results. We did not verify whether the earlier identification of pathogens (and thus earlier and more precise anti-infective therapy) might influence the outcome of the septic patients of this study. However, regarding the available evidence about the benefit of rapid diagnostics in patients with sepsis [3–6], we presume the improved clinical prognosis due to early identification of causative pathogens in our investigation. Furthermore, in this study, we did not perform a cost-effectiveness analysis, including the costs of antibiotic treatment and its side effects, as well as personnel costs and costs for hospital care.

Further steps might be a technical improvement of the methodology, as well as an expansion of the range of primers. The development of fully automated PCR diagnostics might prevent contamination as well as misinterpretation of the results. And, of course, the impact of diagnostic advantages, given by PCR-based molecular amplification techniques, on clinical outcome of anti-infective therapy in patients with sepsis should be demonstrated in a randomized clinical trial.

In summary, several investigations have demonstrated a prognostic improvement in sepsis by timely adequate antibiotic treatment. The use of this time advantage, which is promised by PCR-based techniques, might be an essential step to improve the results of antibiotic therapy in septic patients. Regarding the advantages and limitations of both microbiological culture and PCR procedures, these techniques should be performed in parallel in order to achieve the optimal results in the diagnostics of sepsis.

## Conclusions

The multiplex PCR assay identified more pathogenic microorganisms than the standard microbiological culture technique, when these pathogens were isolated from the blood, but not from the non-blood samples in septic patients. PCR required less time than MC in the identification of causal pathogens from both the non-blood and blood samples. These findings might influence the impact of PCR-based methods in the identification of causal septic pathogens in clinical routine.

## Additional files

Additional file 1:
**Conversion charts.** (PDF 37 kb) Additional file 2:
**Clinical details of 27 septic episodes, where MC technique failed and PCR identified pathogen microorganisms.** (PDF 94 kb) Additional file 3:
**Thirty-five septic episodes with potential time-saving effect of PCR vs. MC technique in identification of pathogen microorganisms.** (PDF 116 kb)
