# Supplementary material for: Prospective comparison of a PCR assay and a microbiological culture technique for identification of pathogens from blood and non-blood samples in septic patients
Source: J Intensive Care. 2015 Nov 21;3:51. doi: 10.1186/s40560-015-0116-1 (PMC4654802; doi:10.1186/s40560-015-0116-1)
Supplement: Additional file 1: — Conversion charts. (PDF 37 kb) [file 40560_2015_116_MOESM1_ESM.pdf]

Supplement 1. Conversion charts.

Chart 1. Scoring table for amount of DNA, yielded from blood samples.

> 0 to 0.2 ng/µl	...	+
0.2 to 0.5 ng/µl	...	++
0.5 to 1.0.ng/µl	...	+++
> 1.0 ng/µl	...	++++

Chart 2. Scoring table for amount of pathogens, yielded from fluid secrets, abscess fluid and smears.

very few – low quantity	...	+
moderate quantity	...	++
many – very many	...	+++
plentiful	...	++++

Chart 3. Scoring table for amount of DNA, yielded from fluid secrets, abscess fluid and smears.

> 0 to 0.9 ng/µl	...	+
1.0 to 9.9 ng/µl	...	++
10.0 to 29.9 ng/µl	...	+++
> 30 ng/µl	...	++++
