# Supplementary material for: Prospective comparison of a PCR assay and a microbiological culture technique for identification of pathogens from blood and non-blood samples in septic patients
Source: J Intensive Care. 2015 Nov 21;3:51. doi: 10.1186/s40560-015-0116-1 (PMC4654802; doi:10.1186/s40560-015-0116-1)
Supplement: Additional file 2: — Clinical details of 27 septic episodes, where MC technique failed and PCR identified pathogen microorganisms. (PDF 94 kb) [file 40560_2015_116_MOESM2_ESM.pdf]

Supplement 2. Clinical details of 27 septic episodes, where MC technique failed and PCR identified pathogen microorganisms*.

Focus of infection	Severity of sepsis	PCT ng/ml	Calculated anti-infective therapy	Sample	PCR result	Clinical decision on further therapy
FUO	SSS	0.1	Piperacillin/Tazobactam, Fluconazole	Blood	*S. aureus* (++)**	Meropenem/Ciprofloxacin because of persistent fever
Abdomen	SSS	38.6	Piperacillin/Tazobactam	Ascites	*E. coli* (+)	Piperacillin/Tazobactam continued
FUO	SS	0.1	none	Blood	*S. epidermidis* (++++)	Piperacillin/Tazobactam after abdominal hematoma excision
Wound	SS	0.3	Cefuroxim	Blood	*S. aureus* (+++), *S. epidermidis* (++)	Meropenem/Ciprofloxacin because of suspected pneumonia
CVC	SSS	0.41	Meropenem, Vancomycin<br>Fluconazole	Blood	*S. aureus* (+++)	Ampicillin/Sulbactam because of suspected CVC infection
Lung	SS	0.1	Gentamicin, Ceftriaxon	BS	*S. aureus* (++)	Meropenem/Ciprofloxacin because of suspected pneumonia
FUO	SS	1.8	Cefuroxim	Blood	*S. epidermidis* (++), *E. faecium* (++)	Meropenem/Ciprofloxacin
FUO	SS	0.14	Ceftriaxon	Blood	*S. epidermidis* (+++)	Death at ICU
FUO	SS	0.1	Clindamycin, Cefotiam	Blood	S. epidermidis (+++)	New CVC because of suspected CVC infection
FUO	SSS	1.8	Piperacillin/Tazobactam	Blood	*S. aureus* (+)	New CVC because of suspected CVC infection
FUO	SSS	2.86	Gentamicin/Ceftriaxon	Blood	*S. epidermidis* (+++), *E. faecium* (++)	Meropenem/Ciprofloxacin, no sepsis focus found
FUO	SSS	2.8	Meropenem/Ciprofloxacin	Blood	*E. faecium* (+)	Meropenem/Ciprofloxacin and Vancomycin
FUO	SS	0.1	Cefuroxim	Blood	*S. epidermidis* (+++)	Imipenem and new CVC
Abdomen	SSS	0.36	none	Blood	*S. aureus* (++++)	Piperacillin/Tazobactam after colon resection
FUO	SS	16.5	Imipenem	Blood	*S. epidermidis* (+++)	New CVC because of suspected CVC infection

FUO	SSS	0.59	none	Blood	*S. epidermidis* (++)	Ampicillin/Sulbactam because of suspected CVK infection
FUO	SS	0.11	none	Blood	*C. albicans*(+++), *S. epidermidis* (++)	Meropenem/Ciprofloxacin
FUO	SSS	0.15	none	Blood	*S. epidermidis* (+), *C. albicans* (+)	None
FUO	SS	0.11	Ciprofloxacin	Blood	*P aeruginosa*(+++), *S. epidermidis* (+)	Ciprofloxacin because of suspected urinary tract infection
FUO	SSS	0.1	Imipenem, Ciprofloxacin, Fluconazole	Blood	*S. epidermidis* (+)	CVC removal
Lung	SS	0.14	Tobramycin, Ceftazidin, Linezolid	BS	*E. faecium* (+++), *C. albicans* (+)	De-escalation to Linezolid due to clinical symptoms
FUO	SSS	0.12	none	Blood	*S. epidermidis* (++)	Portcath removal
FUO	SSS	0.15	Piperacillin/Tazobactam	Blood	*S. epidermidis* (++), *S. aureus* (++++)	CVC removal
FUO	SS	0.28	Piperacillin/Tazobactam, Fluconazole	Blood	*S. epidermidis* (+), K. pneumoniae (+)	Piperacillin/Tazobactam
FUO	SS	0.12	Meropenem, Ciprofloxacin	Blood	*K. pneumoniae* (+)	Piperacillin/Tazobactam
Abdomen	SSS	2.23	Piperacillin/Tazobactam<br>Fluconazole	Blood	P. aeruginosa (+++),E. cloacae (++)	Meropenem, Ciprofloxacin, Fluconazole
Abdomen	SSS	0.43	none	Blood	*S. aureus* (++++), *S. epidermidis* (++),<br>*E. faecium* (+)	Ciprofloxacin, Piperacillin/Tazobactam, Fluconazole

FUO: fever of unknown origin; S: sepsis; SS: severe sepsis; SSS: septic shock; CVC: central venous catheter.

*PCR results were not taken into account to improve the anti-infective therapy of sepsis. Anti-infective therapy was changed only according to clinical course of the disease.

** (+) represents the score for DNA concentration according to conversion chart 1, Supplement 1.
