# Supplementary material for: Prospective comparison of a PCR assay and a microbiological culture technique for identification of pathogens from blood and non-blood samples in septic patients
Source: J Intensive Care. 2015 Nov 21;3:51. doi: 10.1186/s40560-015-0116-1 (PMC4654802; doi:10.1186/s40560-015-0116-1)
Supplement: Additional file 3: — Thirty-five septic episodes with potential time-saving effect of PCR vs. MC technique in identification of pathogen microorganisms. (PDF 116 kb) [file 40560_2015_116_MOESM3_ESM.pdf]

Supplement 3. Thirty-five septic episodes with potential time-saving effect of PCR vs. MC technique in identification of pathogen microorganisms

| Focus of infection | Initial anti-infective (AI) therapy | New AI therapy after MC diagnostics | Causative pathogen (CP) | Date & time of PCR result | Date & time of MC result | Time-saving effect (h) |
|---|---|---|---|---|---|---|
| Lung | Meropenem | Ciprofloxacin/ Ceftazidime | *P. aeruginosa* | 2. Nov. 20:30 | 4. Nov. 8:00 | 35.5 |
| Lung | Ceftriaxone | Meropenem/ Ciprofloxacin | *S. aureus, Enterococi* | 16. Nov. 22:30 | 19. Nov. 8:00 | 17.5 |
| Abscess | Imipenem/ Ampicillin/ Fluconazole | Meropenem/ Ciprofloxacin/ Fluconazole | *P. aeruginosa, E. cloacae, K. pneumoniae* | 26. Nov. 19:00 | 27. Nov. 18:00 | 23 |
| Abdomen | Piperacillin/ Tazobactam | Imipenem/ Ciprofloxacin | *Enterococi* | 11. Jan. 11:30 | 14. Jan. 8:00 | 34.5 |
| Lung | Ceftriaxone | Fluconazole | *C. albicans* | 16. Jan. 16:00 | 18. Jan. 8:00 | 40 |
| Lung | Ceftriaxone | Ciprofloxacin/ Meropenem/ Fluconazole | *E. cloacae, C. albicans* | 14. Feb. 12:30 | 16. Feb. 14:00 | 49.5 |
| Wound | Imipenem | Imipenem/ Vancomycin | *E. faecium, CoNS* | 16. Oct. 18:30 | 17. Oct. 12:00 | 17.5 |
| Abdomen | Vancomycin | Vancomycin/ Ciprofloxacin | *E. cloacae* | 24. Oct. 20:30 | 26. Oct. 16:00 | 43.5 |
| Abdomen | Imipenem | Imipenem/ Fluconazole | *C. albicans* | 29. Nov. 19:30 | 30. Nov. 14:00 | 18.5 |
| Kidney | Cefotiam | AI therapy ceased | *No CP identified* | 14. Oct. 17:00 | 18. Oct. 8:00 | 31 |
| Lung | Clindamycin/ Cefotiam | Meropenem/ Ciprofloxacin | *P. aeruginosa, E. coli, K. pneumoniae* | 21. Jan. 16:00 | 22. Jan. 17:00 | 25 |
| Abdomen | Piperacillin/ Tazobactam | Piperacillin/ Tazobactam/ Vancomycin | *E. faecium* | 22. Sep. 20:00 | 24. Sep. 11:00 | 39 |
| Abscess | Meropenem | Meropenem/ Cefepime | *P. aeruginosa* | 05. Oct. 24:00 | 11. Oct. 12:00 | 24 |
| Abscess | Piperacillin/ Tazobactam | Piperacillin/ Tazobactam/ Fluconazole | *C. albicans* | 21. Nov. 5:30 | 23. Nov. 10:00 | 52.5 |
| CSF | Ampicillin/ Sulbactam | Ampicillin/ Sulbactam/ Vancomycin | *S. epidermidis in CSF* | 13. Feb. 14:30 | 15. Feb. 17:00 | 50.5 |
| Lung | Piperacillin/ Tazobactam/ Ciprofloxacin/ Fluconazole | AI therapy ceased (symptoms due to lung fibrosis and heart insufficiency) | *No CP identified* | 3. Nov. 18:30 | 6. Nov. 13:00 | 66.5 |
| Lung | Ciprofloxacin/ Ceftazidim/ Fluconazole | Imipenem/ Fluconazole | *E. cloacae* | 4. Dec. 24:00 | 6. Dec. 15:00 | 39 |

| Focus of infection | Initial anti-infective (AI) therapy | New AI therapy after MC diagnostics | Causative pathogen (CP) | Date & time of PCR result | Date & time of MC result | Time-saving effect (h) |
|---|---|---|---|---|---|---|
| CVK | Meropenem/ Ciprofloxacin | Ampicillin/ Ciprofloxacin | *E. faecalis* | 21.Nov. 2:30 | 23. Nov. 16:00 | 61.5 |
| Lung | Imipenem/ Vancomycin/ Voriconazol | Imipenem ***(3 MC tests did not yield CP)**** | *No CP identified* | 15. Dec. 24:00 | 20. Dec. 20:00 | 116 |
| Wound | Piperacillin/ Tazobactam | AI therapy ceased ***(2 MC tests did not yield CP)*** | *No CP identified* | 22. Dec. 18:30 | 27. Dec. 8:00 | 109.5 |
| Abscess | Cefotiam/ Clindamycin/ Vancomycin | Cefotiam/ Clindamycin | *S. aureus* | 25. Dec. 14:00 | 27. Dec. 8:00 | 42 |
| Lung | Fluconazole | AI therapy ceased | *No CP identified* | 09. Mar. 12:00 | 11. Mar. 8:00 | 44 |
| Lung | Meropenem/ Ciprofloxacin | Cefotiam ***(first 3 MC tests did not yield CP)*** | *E. coli* | 18. Mar. 11:30 | 24. Mar. 9:00 | 141.5 |
| Abscess | Piperacillin/ Tazobactam/ Clindamycin | Continued ***(first 2 MC tests did not yield CP)*** | *S. aureus* | 23. Sep. 13:30 | 27. Sep. 12:00 | 94.5 |
| Lung | Meropenem/ Ciprofloxacin | continued | *P. aeruginosa* | 15. Oct. 12:00 | 18. Oct. 8:00 | 68 |
| Abdomen | Imipenem | continued | *E. coli, Enterococci* | 25. Oct. 21:30 | 28. Oct. 14:00 | 64.5 |
| Abdomen | Imipenem/ Vancomycin | continued | *Resistant Enterococci* | 11. Nov. 21:00 | 18. Nov. 12:00 | 159 |
| Kidney | Piperacillin/ Tazobactam | continued | *E. coli, E. faecalis in urine* | 20. Nov. 3:30 | 22. Nov. 9:00 | 53.5 |
| Wound | Meropenem/Ampicillin | continued ***(first MC test did not yield CP)*** | *E. cloacae* | 25. Nov. 22:00 | 01. Dec. 8:00 | 130 |
| Abdomen | Imipenem | continued as prophylaxis of necrotizing pancreatitis | *No CP identified* | 21.11., 24:00 | 24.11., 9:00 | 57 |
| Lung | Meropenem/ Ciprofloxacin/ Linezolid | continued | *E. coli, K. pneumoniae* | 20. Dec. 10:30 | 22. Dec. 8:00 | 45.5 |
| CVC | No AI therapy | No AI therapy, clinical situation improved after CVC withdrawal | *P. aeruginosa, E. faecium* | 17. Jan. 20:00 | 19. Jan. 8:00 | 36 |
| CVC | Imipenem/ Ceftriaxone | continued ***(first 3 MC tests did not yield CP)*** | *K. pneumoniae* | 9. Dec. 12:30 | 13. Dec. 9:00 | 92.5 |
| Abdomen | Imipenem/ Ciprofloxacin | continued ***(first 3 MC tests did not yield CP)*** | *Enterococci* | 13. Jan. 8:30 | 17. Jan. 8:00 | 95.5 |
| Lung | Meropenem/ Ciprofloxacin | continued | *E. cloacae* | 17. Mar. 17:00 | 21. Mar. 8:00 | 87 |

MC: microbiological culture; AI: anti-infective; CP: causal pathogen; CVC: central venous catheter; CoNS: *coagulase-negative staphylococci*; CSF – cerebrospinal fluid.

*The explanations for outlier values of potential time-sparing effect are given in bold italics
